# The Seroprevalence of *Toxoplasma gondii* in Wild Boars from Three Voivodeships in Poland, MAT Analyses

**DOI:** 10.2478/s11686-020-00185-3

**Published:** 2020-03-02

**Authors:** Aleksandra Kornacka, Bożena Moskwa, Anna Werner, Piotr Nowosad, Wiesława Jankowska, Aleksandra Cybulska, Anna C. Majewska

**Affiliations:** 1 Witold Stefański Institute of Parasitology, Polish Academy of Sciences, Twarda 51/55, 00-818 Warsaw, Poland; 2 Department of Biology and Medical Parasitology, Faculty of Medicine I, Poznan University of Medical Sciences, Fredry 10, 61-701 Poznan, Poland; 3 Poznan University of Medical Sciences, Fredry 10, 61-701 Poznan, Poland

**Keywords:** *Toxoplasma gondii*, Wild boars, Modified agglutination test, Antibodies

## Abstract

**Purpose:**

The European wild boar (*Sus scrofa*) is a popular game animal species. Its meat, however, can represent a reservoir of dangerous foodborne diseases and can play an important role in the transmission of many pathogens, including *Toxoplasma gondii*, in humans and animals worldwide. The aim of the present study was to determine the presence of antibodies to *T. gondii* in the serum of hunted wild boars in Poland.

**Methods:**

Using the commercial direct agglutination test, 398 serum samples collected during the hunting season 2009/2010 were tested for the presence of *T. gondii* antibodies, and the titre of 40 was considered indicative of *T. gondii* infection in analysed samples.

**Results:**

It was found that nationwide, 37.7% were seropositive to *T. gondii*, although seroprevalence varied from 11.6 to 50% depending on the Voivodeship. Significant differences were observed between the Great Poland and Lubusz Voivodeships and between Great Poland and Warmian-Masurian.

**Conclusion:**

Serological test indicated widespread exposure to *T. gondii* by wild boar; therefore, consumption of raw or undercooked game meat of infected animals can carry a significant risk of *T. gondii* infection.

## Introduction

The wild boar (*Sus scrofa*) is one of the most widely distributed large mammals across the territory of Poland and Europe [24]. The species engages in destructive behaviour and causes damage to agricultural fields, for this *S. scrofa* is considered one of the hundred most harmful invasive species [25].

Because of their behavioural characteristics and omnivorous eating habits (consuming mainly plant matter but also live and dead animals), the wild boar is a potential carrier of viral, bacterial, and parasitic diseases such as porcine circovirus, tuberculosis, trichinellosis and toxoplasmosis that can affect livestock, wildlife and humans [28].

In accordance with FAO, *T. gondii* it is the fourth most important foodborne parasite (the first among the protozoa) worldwide [12]. The parasite infects most warm-blooded animals including humans. Wild and domestic felids are the definitive hosts (excrete the oocysts in their faeces), while many mammals and avian species are intermediate hosts [10]. After primary infection, a single cat may shed more than 100 million oocysts into the environment [37]. Under environmental conditions with sufficient aeration, humidity, and warm temperature, oocysts may sporulate and become infective in less than 1 day. Human toxoplasmosis is usually acquired by three principal routes: (1) ingestion of oocysts in material contaminated by faeces of infected, shedding felines, (2) ingestion of *T. gondii* cysts in raw or undercooked meat of infected animals, or (3) transplacental transmission of the parasite tachyzoites from a parasitemic host to its foetus. Although *T. gondii* infection is usually asymptomatic in humans, it can cause considerable morbidity and mortality especially in immunocompromised individuals. Pregnant woman can congenitally infect the foetus, tachyzoites of *T. gondii* are capable to pass through placenta, and the damage caused by this parasite to the unborn child is often more severe the earlier in pregnancy the transmission occurs [19].

Over the last decade, food habits have been changing constantly, and as wildlife meat is widely regarded as being healthier, consumption of game meat has become more popular. Meat from infected animals is considered the most important source of *T. gondii* human infections [17]. Also, the European Food Safety Authority (EFSA) reports that more than 50% of all human toxoplasmosis cases are foodborne [11, 35].

In addition, the disease burden of human toxoplasmosis on healthcare systems in Western countries is ranked second among the most common foodborne pathogens for causing death [11]. However, in many countries, no data are given regarding the prevalence of *T. gondii* infection in human or animal populations, which suggests that the disease is still widely underestimated and scarcely investigated [38].

Since tissue cysts are not visible on postmortem inspection, meat of infected animals represents a source of infection for man [34]. The risk of infection is further increased by the consumption of the hunted wild boar by hunters and their families, or the use of meat of infected animals in products intended for the local market. Where hunters leave animal carcasses or leftovers of deer, boars or foxes out in the field, there is a chance that wild boars scavenge on them, leading to further chances of infection with *Toxoplasma*.

The aim of the present study was to determine the occurrence of antibodies to *T. gondii* in wild boars from three Voivodeships in Poland by modified agglutination test (MAT).

## Materials and Methods

**Fig. 1:**
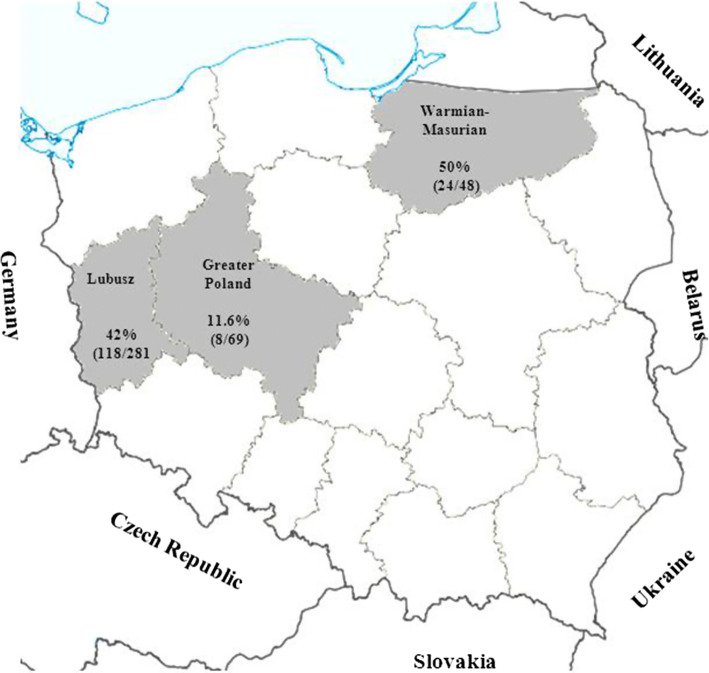
Map showing the sampling areas and the seroprevalence of *Toxoplasma gondii*

Blood samples were collected from 398 wild boars in 2009–2010 during hunting season in Poland, supervised by the Polish Hunting Association. The study was performed in three Voivodeships: Warmian–Masurian (*n* = 48), Greater Poland (*n* = 69), Lubusz (*n* = 281) (Fig. 1). Blood was collected in sterile tubes directly from the thoracic cavity upon evisceration. The sera were separated and kept at − 20 °C until analysis. No data about sex and age was available.

### Area of the Study

Warmian–Masurian Voivodeship is located in north-eastern Poland, the area of 24,192 km^2^ consists mainly of forests, lakes and agricultural fields and farms. According to Polish Institute of Meteorology and Water Management—National Research Institute reports, the overall precipitation is 600.3–729.0 mm, average annual temperature is 6.8–6.2 °C with humidity about 83.0–84.3%.

Lubusz Voivodeship is the smallest Polish providence in western Poland, with overall area of 13,988 km^2^. The region is mainly flat, with many lakes and woodlands, categorised as a one of the warmest in Poland. The overall temperature is 9.2 ÷ 7.9 °C with average annual humidity around 78.8 ÷ 78.4% and overall precipitation 585.8 ÷ 686.1 mm. One of the biggest Voivodeships in Poland is Great Poland Voivodeship, located in west-central Poland. It has an area of 29,826 km^2^ and agriculture and touristic are two main branches of this region. The Great Poland Voivodeship is influenced by oceanic air masses that affect the mildness of the climate, the overall temperature reaches 9.2 ÷ 7.7 °C, and the average precipitation varies from 585.5 mm to 715.3 mm with humidity about 77.5 ÷ 79.6%.

### Modified Agglutination Test (MAT)

The sera were tested for the presence of *T. gondi*-specific IgG antibodies using MAT (Toxo-Screen DA, BioMérieux SA, France). Equal volumes (25 µl) of the serum sample were added into each well, both the negative and positive controls provided in the kit were included in each test. The dilutions that formed a foil or mesh over at least 50% of the base of the microplate well were considered as seropositive, and dilutions that formed a compact button or occupied up to 50% of the base were considered seronegative. A titre of 40 was considered indicative of *T. gondii* infection in the sample (the minimal titre for a positive result in this kit was established as greater or equal to 40 [36].

### Statistical Analysis

The statistical analyses were performed using the StatPages® free statistical software. A chi-square test was used. The differences were statistically significant when *p* < 0.05. To calculate the seroprevalence and the 95% confidence intervals, a binomial confidence intervals calculator was used (https://statpages.info/confint.html).

## Results

Antibodies to *T. gondii* were detected in 150/398 (37.7%, 95%CI 32.9%–42.6%) wild boar serum samples. The seroprevalence varied between Voivodeships. Among Greater Poland wild boars, the seroprevalence was 11.6%, Lubusz 42% and Warmian-Masurian 50%. The statistical analysis showed that there are significant differences between the Great Poland and Lubusz Voivodeships and also between Great Poland and Warmian-Masurian.

Among the positive results, 80.6% of antibodies were detected in titre range 40–180, while 16.7% were found in 540–4000 and 2.7% in the ≥ 6000 range (Table 1).

**Table 1:** Titre distribution among examined wild boars from three studied Voivodeships

Voivodeship	Titre								
	40	60	180	540	1620	4000	6000	18,000	Total *N*/*n*
Great Poland	2	3	1	0	1	1	0	0	8/69
Lubusz	41	38	17	11	4	4	2	1	118/281
Warmian-Masurian	8	9	2	2	0	2	1	0	24/48
Total	51	50	20	13	5	7	3	1	150/398

*N* number of positive samples, *n* number of examined samples

## Discussion

Both livestock and wild boars can act as intermediate host for *T. gondii*. These species may act as one of the sources for parasite transmission to humans [16]. Many epidemiological studies have found an association between the consumption of undercooked or raw meat and *T. gondii* infection in humans [5, 8, 10].

*T. gondii* infection has been reported many times in Poland among farm animals; however, the current status of infection among wildlife is unknown [36, 40]. Earlier studies conducted by Bień et al. [4] revealed 29.8% seroprevalence among wild boar samples from Lublin Voivodeship and 34.45% among those from Warmian-Masurian. A similar seroprevalence (21.1%) was identified in Lublin Voivodeship by Sroka et al. [36]; however, the number of examined samples was low (*n* = 52). Total *T. gondii* seroprevalence in wild boars reported in the present study (37.7%) is comparable with that obtained by Witkowski et al. [40] (37.6%); however, the precise origin of the samples in their study is not stated.

Lower seroprevalences have been detected in bordering countries, with 25% observed in Germany [27], 8.1% in Slovakia [2] and 26.2% in the Czech Republic [3]. Interestingly, a rise in seroprevalence has been observed in Slovakia and the Czech Republic over the following years, reaching 39.7% and 40%, respectively [30, 31].

Jokelinen et al. [18] used MAT to examine 471 individual wild boar samples submitted by hunters in Estonia, the results showed that 113 (23.99%) animals had antibodies against *T. gondii*. Seroprevalence presented by Jokelinen et al. is lower comparing to our results, however, it has been proven that the choice of diagnostic method can influence the heterogeneity of results. A higher seroprevalence of *T. gondii* was observed in studies employing ELISA (enzyme-linked immunosorbent assay) or MAT than in those which used LAT (latex agglutination test). Using ELISA, the highest prevalence of antibodies to *T. gondii* in Europe has been demonstrated in wild boars from Sweden (50%) and Romania (56.7%) [15, 39]. A relatively high prevalence was reported using MAT in studies conducted by Richomme et al. [32] in France (40.4%) and in Spain (43.5%) [7]. In contrast, studies performed in China on serum samples using LAT returned a prevalence of 7.2% (27/377) [26]. Several studies have compared these immunological tests [13, 20, 33].

Seroprevalence to *T. gondii* in animals depends not only on serological tests performed but also on several other factors such as presence of felids, climatic conditions, animal species examined. In general, the highest values of seroprevalence of *Toxoplasma* have been found in game of area with relatively high humidity [14], with an environmental conditions that favour the survival of *Toxoplasma* oocysts. In our study the highest seroprevalence was observed in Warmian-Masurian Voivodeship with relatively high humidity. The area consist mainly of forests and small villages with many farms. The presence of feral cats and relatively good climate conditions gives opportunity for *T. gondii* oocysts to persist in the environment.

Consumption of raw or undercooked meat of infected animals is the main route of infection in humans in Europe, representing 30–63% of all infections, depending on the country [8, 10]. In addition, the EFSA [11] has recommended monitoring game meat for the presence of *T. gondii*. To address these high infection rates, simple post-harvest procedures have been suggested for killing *T. gondii* cysts in meat intended for consumption, such as freezing, heating, irradiation or high-pressure treatment [21]. Interestingly, Kijlstra and Jongert [22] suggest that frozen meat is safe for consumption, with regard to *Toxoplasma*. They recommend that consumers should freeze the meat for at last 2 days at temperatures below − 12 °C, or properly cook it, to prevent cross-contamination while preparing meat in the kitchen.

Recently, game has become more popular as a meat source for humans in many countries and *S. scrofa* is the most important game animal species in the world [29]. In addition, wild boar hunting is a popular recreational sport and a population control method supported by wildlife agencies. Despite intensive hunting, the wild boar population of Poland continues to grow. According to data published by the Central Statistical Office of Poland, their number rose from 118 300 in 2000 to 264 000 in 2015, and the number of hunted wild boar rose threefold during this time. It is important to note that Poland is one of the leading European exporters of game: in 2014, 3363 tonnes of wild boar meat was exported, representing over 30% of all exported game meat [23]. It was also found that meat products are often produced by combining meat from different animal species, which significantly increases the risk of infection [1, 6].

Poland has a strong tradition of consuming wild boar meat. Although the meat is usually heated long enough to inactivate *T. gondii*-infective stages*,* some specific dishes or products, such as smoked sausage, may not be cooked sufficiently, or at all. Additionally, tasting meat during cooking or poor kitchen hygiene is the main risk factors for human infection. The risk of infection is also exacerbated by the fact that no obligation exists for screening meat for the presence of *T. gondii* during meat inspection.

Wild boar meat is a potential threat not only for humans, but also for other animals. Wild boar infected by *T. gondii* migrates for long distances, and can serve as sources of infection for other scavenging and omnivorous animals through the consumption of infected viscera and carcasses left by hunters [9].

To conclude, our findings indicate a relatively high seroprevalence of *T. gondii* among wild boars originating from three Voivodeships of Poland. Results highlight the zoonotic potential of wild boars, and emphasise that game meat should not be neglected as a significant source of *T. gondii* infection for humans and animals. Both farmed and wild animals living close to human settlements may be exposed to oocysts shed from domestic cats, either directly or via contaminated water. We cannot neglect that poor management and lack of adequate hygiene of hunters handling meat, organs and carcasses of wildlife may play an ecological role in the transmission of *T. gondii* infection. Hunters should rather incinerate carcasses and residuals of hunting to avoid risk of infection to omnivores and carnivores—especially felids that may contaminate environment with oocysts which in turn may infect wild game that will later be consumed by humans. It is recommended that surveillance programmes should be implemented in Europe.

## Data Availability

Data supporting the conclusions of this article are included within the article.
